# Operationalizing Engagement With an Interpretation Bias Smartphone App Intervention: Case Series

**DOI:** 10.2196/33545

**Published:** 2022-08-17

**Authors:** Ramya Ramadurai, Erin Beckham, R Kathryn McHugh, Thröstur Björgvinsson, Courtney Beard

**Affiliations:** 1 Department of Psychology American University Washington, DC United States; 2 Cognition and Affect Research and Education Lab McLean Hospital Belmont, MA United States; 3 Division of Alcohol, Drugs, and Addiction McLean Hospital Harvard Medical School Belmont, MA United States; 4 Behavioral Health Partial Hospital Program McLean Hospital Harvard Medical School Belmont, MA United States

**Keywords:** engagement, mental health apps, cognitive bias modification, human support, mobile health, mHealth, mobile phone

## Abstract

**Background:**

Engagement with mental health smartphone apps is an understudied but critical construct to understand in the pursuit of improved efficacy.

**Objective:**

This study aimed to examine engagement as a multidimensional construct for a novel app called *HabitWorks*. *HabitWorks* delivers a personalized interpretation bias intervention and includes various strategies to enhance engagement such as human support, personalization, and self-monitoring.

**Methods:**

We examined app use in a pilot study (n=31) and identified 5 patterns of behavioral engagement: consistently low, drop-off, adherent, high diary, and superuser.

**Results:**

We present a series of cases (5/31, 16%) from this trial to illustrate the patterns of behavioral engagement and cognitive and affective engagement for each case. With rich participant-level data, we emphasize the diverse engagement patterns and the necessity of studying engagement as a heterogeneous and multifaceted construct.

**Conclusions:**

Our thorough idiographic exploration of engagement with *HabitWorks* provides an example of how to operationalize engagement for other mental health apps.

## Introduction

### Background

Over the past 2 decades, the number of available mental health smartphone apps has grown to well over 10,000 [1]. Compared with the number of apps available, research testing the efficacy of apps is extremely limited [2,3]. However, a growing body of research supports the clinical benefits of some mental health apps for a range of emotional disorders (anxiety and depression [4], anxiety [5], depression [6], schizophrenia [7], and alcohol [8]), particularly when app use is supported by some level of human coaching [9,10].

A critical challenge to realizing the potential of mental health apps is attrition; app use has been found to decline significantly after the first 2 weeks [11], and a recent review of health app use from >100,000 users found that the average period of use was just 5.5 days [12]. Mental health app users rarely complete the “full course” of the app intervention [13]. There are many possible explanations for declining app use; for example, users may have “gotten what they need” [4], or the app has “lost its novelty” [14]. Intervention fatigue (emotional or cognitive weariness attributed to the intervention) [15], lack of accountability, and low alliance with app interventions have been highlighted as the reasons for disengagement [16]. Furthermore, the presence of technical issues [17] or general unhappiness with app features are obvious reasons for discontinued use and emphasize the need to incorporate user input into the app design process [18].

Although there is an implicit assumption of a meaningful relationship between app use and benefit, the relationship between app use and clinical outcomes is complex. Greater app use has not consistently been associated with better clinical outcomes (eg, Lin et al [19] and Bakker et al [20] found association between app use and clinical outcomes, and Graham et al [4] found no association between app use and clinical outcomes). Thus, researchers have called for more attention to engagement [1,21], suggesting that the way in which people use and relate to (ie, connect with and enjoy) the app may have important implications. This user-app relationship occurs both during and outside of actual app use and may be central to app efficacy [22]. Although systematic research on the most effective methods to enhance engagement is lacking, a recent review identified a broad range of factors that may facilitate engagement, including increases in insight, sense of control over one’s mental health challenges [23], and human connections incorporated into the intervention [11,24].

Although there are many definitions of engagement, most concur on its multifaceted and dynamic nature [23,25] suggesting that it subsumes the extent of intervention use (amount, frequency, and duration), as well as subjective experience (attention, interest, and affect) with the intervention [26]. Nahum-Shani et al [27] integrated theories of engagement across disciplines (ie, education, industrial or organizational psychology, and computer science) and suggested that engagement may be best thought of as “energy investment involving physical, affective, and cognitive energies directed toward a focal stimulus or task.” Recent examinations of engagement have indeed focused on a tripart model: behavioral (physical involvement with the intervention), cognitive (thinking about, attending to, and processing the intervention), and affective (emotional response to the intervention) [23,27-29]. These 3 domains are distinct; an individual can enjoy an intervention (affective) but not complete the suggested amount of use (behavioral), or they can complete intervention sessions (behavioral) and not make connections between the app and their life (cognitive). Nahum-Shani et al [27] asserted that engagement is a *state* that waxes and wanes because of a variety of internal and external factors [30] rather than a relatively stable construct [28,31].

### This Study

In this study, we aimed to operationalize the model of engagement by Nahum-Shani et al [27] for a novel mental health app called *HabitWorks*. We developed *HabitWorks* to provide support during the critical transition between acute psychiatric care and outpatient treatment, a time of high risk for symptom deterioration, rehospitalization, and treatment disengagement [32]. *HabitWorks* was initially developed for patients receiving cognitive behavioral therapy (CBT) skills–based partial hospital care and was designed to augment treatment by facilitating the practice of cognitive therapy skills, to promote skill practice in the postacute period, and to ease the transition back into community treatment [33].

*HabitWorks* delivered a personalized interpretation bias intervention, as well as self-monitoring. This intervention was designed to promote an adaptive interpretive style, as the tendency to interpret ambiguous situations negatively (or not interpret them positively) plays an important role in the maintenance of most emotional disorders [34]. This type of intervention reliably improved interpretation bias and, in some cases, led to improved clinical symptoms [35,36]. The interpretation bias exercise was framed as a way for participants to practice catching themselves when jumping to negative conclusions, ultimately fostering healthier mental habits. The symptom-monitoring component was presented as a way of raising awareness about mood fluctuations.

In a small pilot study, *HabitWorks* was feasible and acceptable for a transdiagnostic sample of patients attending a partial hospital program [33]. Qualitative feedback revealed that participants enjoyed using the app and related the content to their daily lives. Although adherence was excellent during acute care (ie, 78.6% met the 5-session benchmark), similar to many apps [11], use throughout the month after discharge decreased over time (ie, based on the 3-session weekly adherence benchmark, weeks 1-3: approximately 33% adhered; week 4: approximately 0% adhered) [33]. Increasing app use during the month following discharge is likely to be vital to the efficacy of *HabitWorks*, as similar cognitive bias modification interventions have been found to be most effective with practice and repetition [36,37]. Consequently, we made several refinements to the app to enhance engagement during the postdischarge period.

This study aimed to (1) present an operationalization of engagement with *HabitWorks* based on the 3-facet model, (2) identify patterns of behavioral engagement with *HabitWorks* during the month after discharge, and (3) present case examples to illustrate 5 distinct patterns of behavioral engagement. The identification of engagement patterns was based solely on use because of the objectivity of the measurement, precedent regarding the way in which engagement patterns have been categorized in larger studies [11,38] and our project’s a priori determination of adherence. Although we primarily focused on behavioral engagement for pattern categorization, we also explored indicators of affective and cognitive engagement. Research indicates that presenting only behavioral outcomes may be simplistic and fail to fully capture engagement as a construct [27]. Examining affective and cognitive engagement is crucial for developing a more thorough and nuanced understanding of the way people interact with apps. An idiographic approach was preferred to achieve a rich understanding of patterns of engagement [39,40] with this new app and as research on other apps has highlighted the heterogeneity in the preference of app features [26]. Exploring individual patterns of engagement with *HabitWorks* may inform further tailoring of the app to enhance its efficacy for high-risk populations, as well as inform the development of similar types of mental health apps.

## Methods

### Participants and Setting

This study included 31 participants who were randomly assigned to *HabitWorks* in a pilot randomized controlled trial (RCT; Table 1 provides the demographic characteristics of participants). Participants were recruited from a partial hospital program at McLean Hospital in Belmont Massachusetts, which provides intensive, CBT-based, transdiagnostic treatment. Inclusion criteria included at least moderate symptom severity at admission (>9 on the Patient Health Questionnaire-9 [41] or Generalized Anxiety Disorder-7 [42]), at least a minimal level of interpretation bias (<80% accuracy on the Word Sentence Association Paradigm [WSAP]; [43]), having an Apple iPhone (*HabitWorks* was not compatible with Android), and willingness to sign a release form to communicate with outpatient providers in case of any safety concerns.

**Table 1:** Full sample demographics (N=31).

Characteristics			Values
Age (years), mean (SD)			29.2 (10)
**Gender, n (%)**			
	Nonbinary transmasculine	1 (3)	
	Woman	19 (61)	
	Man	11 (36)	
**Sexual identity, n (%)**			
	Queer	1 (3)	
	Bisexual	3 (10)	
	Gay or lesbian	2 (7)	
	Heterosexual	25 (81)	
**Ethno-racial identity, n (%)**			
	Do not know	1 (3)	
	Asian and White	3 (10)	
	Asian	2 (7)	
	Hispanic or Latinx	2 (7)	
	Non-Hispanic White	24 (77)	

Exclusion criteria included current mania, psychosis, or severe clinical acuity, as judged by clinic staff, which would impair the understanding of consent and research procedures. Forgeard et al [44] provided a thorough overview of the partial hospital program, and Beard et al [33] provided the description of eligibility for the *HabitWorks* study. Eligible participants provided informed consent to participate in the study procedures as an augmentation to their care as usual. The 5 case examples chosen from the larger sample (N=31) have been masked such that they include no identifiable information, and all demographic data (ie, diagnosis and occupation) have been changed.

### Ethics Approval

This study was approved by the Mass General Brigham Institutional Review Board (2018P000252).

### HabitWorks Intervention

*HabitWorks* delivered a personalized, transdiagnostic interpretation bias intervention. The app was developed in consultation with content experts and clinic directors for implementation strategy. Given the importance of user involvement in the development process [1], a patient advisory board and open trial participants provided critical feedback throughout the development process, informing modifications to the app and methods to enhance engagement [33]. Table 2 provides a detailed list of *HabitWorks* features and prior evidence supporting their usefulness.

**Table 2:** Features of *HabitWorks* and strategies used to enhance engagement.

Feature or strategy	Empirical support	What does this look like in *HabitWorks*?
Human support	[11,39,45-50]	App use was guided during acute care as support staff checked in with participants daily or less frequently if preferred. Postdischarge support was continued through weekly email check-ins.
Customization and notifications	[51-53]	Participants were prompted to schedule 3 exercise sessions per week in the month after discharge and were then sent push notifications at the scheduled times. Exercise scheduling was customizable such that participants could schedule and change exercise session timing, promoting participants’ sense of control and feasibility to use in the context of the participant’s busy life.
Personalization	[45,54]	Increased relevancy of HabitWorks by only offering it to those who demonstrated at least a minimal level of interpretation bias. Participants completed personalization checklists assessing demographic characteristics and worry domains (eg, social situations, panic symptoms, and relationships). The app algorithm then selects relevant word-sentence pairs (see the study by Beard et al [33] for checklists).
Novelty	[55,56]	HabitWorks presented variations of the interpretation bias exercise in format and length through the “level up” and bonus functions. When participants reached 90% accuracy, they progressed to the next out of 10 levels, which featured increasingly positive interpretations and introduced novel word-sentence pairings [33]. The app presented 17 randomized encouraging GIFs, such as a celebrity giving a thumbs up, at the end of each exercise session.
Mood and tracking features	[45,50,57-60]	Participants completed mood surveys prompted by the app weekly and self-initiated surveys as desired. HabitWorks included progress graphs of mood check-in data, as well as exercise performance. The exercise graphs depicted changes in reaction time and interpretation accuracy over time.
Habitdiary	[61-63]	The Habitdiary asked participants to reflect on their week and record instances in which they found themselves jumping to negative conclusions or noticed changes in their thinking or behavior. Participants were prompted to complete entries once weekly during check-ins and could also initiate additional entries as desired.
Feedback	[54,60]	HabitWorks provided feedback during the exercise to participants immediately following each trial based on the accuracy of their responses (ie, “Correct!” Or “Try Again!”), as well as at the end of each exercise on overall reaction time, accuracy, and percentage improvement (see the study by Beard et al [33] for a description of feedback). HabitWorks provided PHQ-9^a^ and GAD-7^b^ scores.
Privacy and data security	[18,64-66]	Users required a unique passcode to access HabitWorks. HabitWorks enabled touch ID to access the app and ensured thorough understanding of participant rights, data collected, data storage techniques, and data uses by going over consent documentation and storing this document within the app.

^a^PHQ-9: Patient Health Questionnaire-9.

^b^GAD-7: Generalized Anxiety Disorder-7.

**Figure 1:**
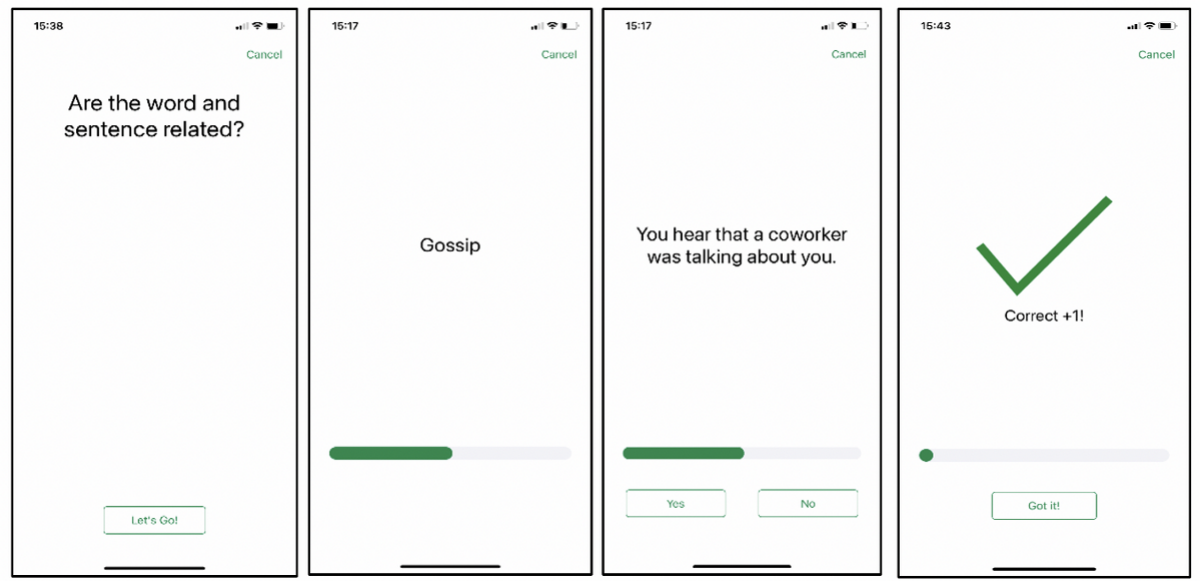
Supplemental screenshots of a *HabitWorks* exercise trial.

The interpretation bias exercises were based on the WSAP [43,67]. At the onset of the exercises (Figure 1 provides screenshots), participants were instructed to imagine themselves in each of the upcoming situations. Next, a word was presented that represented a positive (*funny*), neutral (*toast*), or negative (*embarrassing*) interpretation of an ambiguous situation that followed (*during your speech at the wedding, you notice people in the audience laughing*). Participants clicked “yes” or “no” on their phone screen, indicating whether they believed the word and sentence were related. Next, they were presented with corrective feedback (ie, “Correct!”) based on the accuracy of their responses. In this task, endorsing neutral or positive interpretations and rejecting negative interpretations were considered as accurate responses.

*HabitWorks* delivered several versions of the WSAP, varying by the length and order of the stimuli. Each exercise after discharge comprised 50 trials. Additional variations of the task included the following: (1) a *reverse exercise* (the sentence was followed by the word), (2) a *bonus session* (only 30 trials), and (3) a *habit test* (personalized assessment version of the task in which there was no corrective feedback).

Participants were asked to use the app daily during acute care, with support from bachelor’s degree–level research staff as desired. This report focuses on engagement during the month following discharge, during which participants were asked to complete exercises 3 times per week independently, as well as a weekly in-app check-in that included a mood check-in (ie, depression and anxiety scores) and the habit test. During this postdischarge period, participants continued to be supported via weekly email check-ins from the staff. Participants were asked to complete assessments after treatment (1 week after discharge) and after 1 month (1 month after discharge). Participants were compensated US $100 for completing the study assessments but were not compensated for their app use.

### Measures

#### Overview

**Figure 2:**
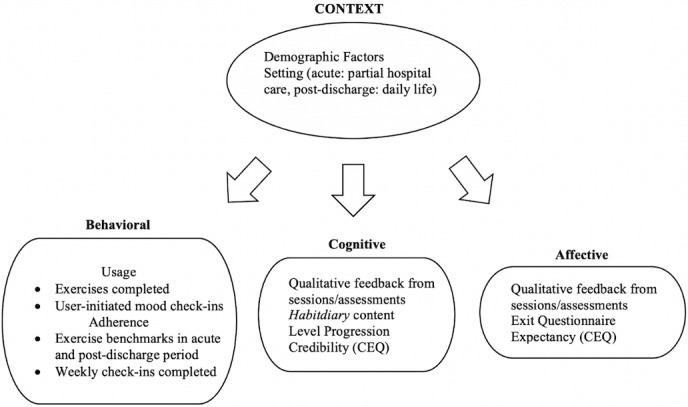
Operationalization of engagement in HabitWorks based on the visual model used by Nahum-Shani [27] and created by Appleton et al [69]. CEQ: Credibility and Expectancy Questionnaire.

Measures were administered via the *HabitWorks* app, as well as on the web using REDCap (Research Electronic Data Capture; Vanderbilt University) [68]. Figure 2 [27,69] shows the indicators used for the measurement of each engagement facet. Of note, although some indicators of engagement were planned a priori (eg, number of exercises completed and affective ratings on exit questionnaire), others were selected post hoc based on available data from the RCT (eg, Habitdiary entries).

#### Behavioral Engagement

##### Use

We calculated the number of exercises completed per week, number of Habitdiary entries completed, and number of self-initiated mood surveys.

##### Adherence

Adherence to the protocol was defined as the completion of the suggested 12 exercises and 4 weekly check-ins during the 1-month postacute phase of the study.

#### Cognitive Engagement

##### Credibility and Expectancy Questionnaire (Credibility Only)

After the first session of *HabitWorks*, the participants were asked to complete the Credibility and Expectancy Questionnaire (CEQ) [70]. The CEQ is a widely used 6-item self-report measure with items that load on 2 factors: credibility (items 1-3) and expectancy (items 4-6). A rating scale of 1 (*not at all*) to 9 (*completely*) or 0% to 100% is used for each question, depending on the question content. The credibility items from the CEQ assess how logical the participants *believe* the intervention to be. We examined the initial ratings of credibility as a measure of early-stage cognitive engagement with the intervention.

##### Habitdiary

**Figure 3:**
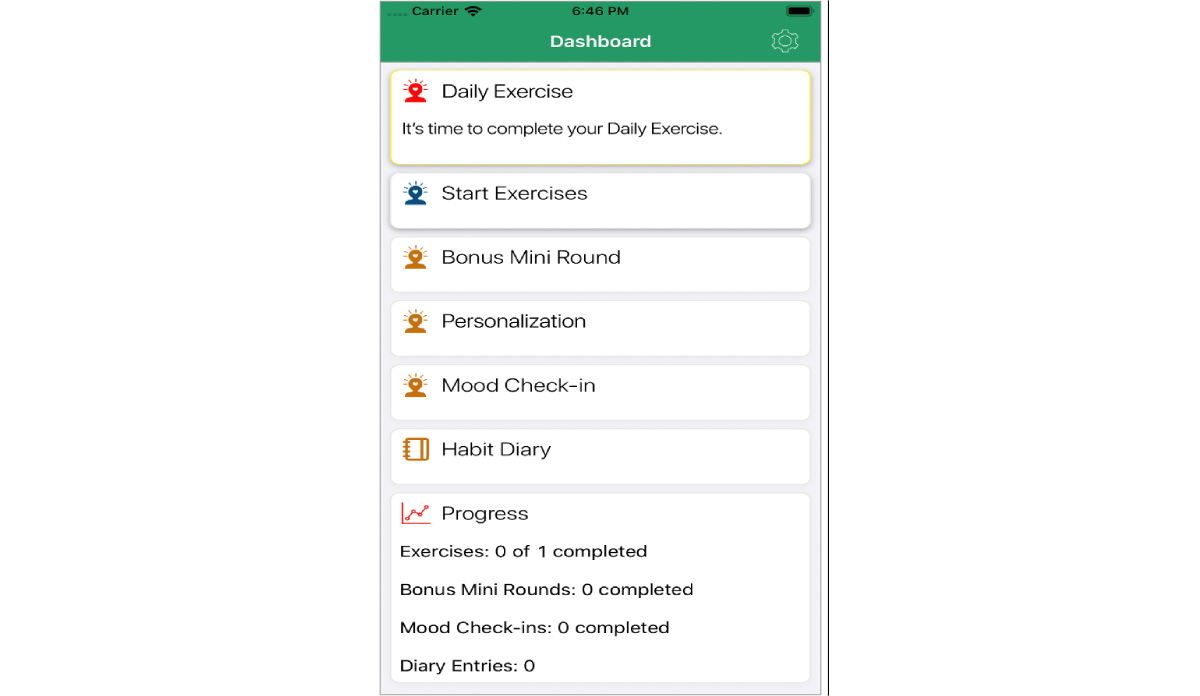
Supplemental screenshot of the *HabitWorks* dashboard.

Participants were asked to complete free-response diary entries weekly during the 1-month postdischarge phase and were able to initiate additional entries as desired from the dashboard of the app (Figure 3). The content of the entries was coded as an indicator of the degree to which participants applied the app content to their lives or used the feature as a free-response diary.

##### Level Progression

The participants progressed through a series of 10 levels in the *HabitWorks* app based on exercise performance [33]. As participants progressed through the levels, they were presented with increasingly positive stimuli to endorse compared with more neutral stimuli at the beginning. As such, the achieved levels corresponded with mastery of the task and the content received (ie, more positive stimuli). To progress from one level to the next, the participants had to achieve 90% accuracy in their exercise. Importantly, an accuracy score of 70% on the assessment version of the WSAP (ie, no corrective feedback) reflects a healthy, nonanxious interpretation style [43]. We examined the final achieved level as a marker of cognitive engagement with the app.

##### Qualitative Feedback

Participants were asked to provide feedback on the *HabitWorks* app verbally during each assessment time point and during weekly check-ins conducted via email. In addition, qualitative interviews were conducted at the 1-month assessment by the senior author (CB). Although qualitative interviews were not initially intended to assess engagement, several prompts (ie, “Do you feel like anything’s changed with you since you started the *HabitWorks* app?” and “Are you thinking about yourself or other people differently?”) reflect our theoretical understanding of cognitive engagement (Multimedia Appendix 1 provides a full measure). Feedback from assessments and sessions underwent rapid coding qualitative analysis [71] by the first (RR) and second (EB) authors to identify predominant themes related to the ways in which participants connected the app to their other treatment or daily life. These data were subsequently used as indicators of cognitive engagement.

#### Affective Engagement

##### Exit Questionnaire

We administered a self-reported measure of satisfaction [35]. This exit questionnaire prompted participants to rate how helpful, relevant, user-friendly, and satisfying they found *HabitWorks* on a scale with options ranging from 1 (*completely disagree*) to 7 (*completely agree*; Multimedia Appendix 1 provides the full measure).

##### Qualitative Feedback

Several items (ie, “What did you think about the *HabitWorks* app?”; “What did you find beneficial?”; “What was not helpful?”) included in the qualitative interview reflect our theoretical understanding of affective engagement (Multimedia Appendix 1 provides the full measure). This qualitative interview along with assessment feedback underwent rapid coding qualitative analysis (described previously). Themes and feedback that were identified as reflective of their experience (eg, enjoyment and irritation) using *HabitWorks* were used as indicators of affective engagement.

##### CEQ (Expectancy Only)

The expectancy items assessed how participants *feel* regarding the intervention’s potential to reduce their symptoms. We explored the ratings of expectancy toward *HabitWorks* as a measure of early-stage affective engagement with the intervention.

## Results

### Behavioral Engagement Patterns Overview

App use data were passively collected within the app and stored on a secure REDCap server. Upon study completion, data were exported and aggregated by participants for the following variables: type of use, date, and content related to use (eg, accuracy score for exercises, mood symptom score, and Habitdiary content). Use during the month after discharge was focused on, as many factors (ie, insurance, clinical acuity, and logistics) affected the length of stay in acute care, making comparisons of use during acute care challenging. We calculated the following summary variables for the month after discharge: number of exercises completed per week, number of weekly check-ins completed (of 4), number of Habitdiary entries completed, and number of user-initiated mood surveys completed.

After a thorough visual inspection of the data, the first (RR), second (EB), and last (CB) authors discussed and came to a consensus to identify 5 patterns of engagement in the month after discharge. The 3 authors then independently categorized participants into one of the 5 use patterns: consistently low (0-2 exercises per week; 5/31, 16%), adherent (9-15 exercises during month; 14/31, 45%), drop-off (adherent initially, then dropout; 2/31, 6%), high diary (adherent plus >2 diaries per week; 3/31, 10%), and superuser (>16 exercises during month, 7/31, 23%). We then selected the cases that represented each engagement pattern. Table 3 provides a summary of participant engagement indicator data.

**Table 3:** Summary of participant engagement.

Facet and indicator		Participant A (consistently low)	Participant B (adherent)	Participant C (drop-off)	Participant D (high diary)	Participant E (superuser)
**Behavioral**						
	Exercises during 1 month after discharge (suggested 12), n (%)	4 (33)	13 (108)	13 (108)	10 (83)	60 (500)
	Number of Habitdiaries	4	4	6	11	17
	Weekly check-ins (suggested 4), n (%)	3 (75)	3 (75)	2 (50)	4 (100)	4 (100)
	Number of user-initiated surveys 1 month after discharge	3	3	7	1	22
**Cognitive**						
	1 (not at all) to 9 (completely)	7	6	5	9	7
	Credibility: useful—1 (not at all) to 9 (completely)	6	7	3	5	5
	Level completion by 1 month (out of 10 levels), n (%)	4 (40)	8 (80)	10 (100)	1 (10)	10 (100)
	Habitdiary content	Relationship functioning, eating behaviors and symptoms, and interpersonal conflict	Dating, current treatment, general mental health status, and awareness of symptom improvement	Symptom improvement and current treatment, social functioning, work, and COVID-19–related worries	Free-response record (ie, monitored with timings): sleep, food, symptoms, and medication	Worries about the future, romantic relationships, family, and health
**Affective**						
	Expected improvement (%)	80	30	10	70	30
	Exit questionnaire: 1 (completely disagree) to 7 (completely agree), mean (SD)	6.6 (0.55)	6 (0.71)	N/A^a^	5.6 (0.55)	6.6 (0.55)

^a^N/A: not applicable.

### Participant A: “Consistently Low”

Participant A was a college student with a primary diagnosis of bipolar disorder. Participant A maintained a low level of activity in the app throughout the month after discharge and completed the 1-month follow-up assessment.

#### Behavioral Engagement

During the month after discharge, participant A completed 75% (3/4) of the weekly check-ins, as well as 3 self-initiated mood check-ins. Exercise completion during the month after discharge was low (ie, 4), reflecting low and sporadic use: participant A completed 1 exercise in week 1, a total of 2 exercises in week 2, no exercise in week 3, and 1 exercise in week 4.

#### Cognitive Engagement

At baseline, participant A’s cognitive engagement, assessed by credibility ratings (out of 9=“completely”), was good (treatment logicality=7 and usefulness of treatment=6). Participant A completed 4 Habitdiary entries that covered several themes such as relational functioning and interpretations (ie, family, social, and romantic relationships), eating-related symptoms, and interpersonal conflict. Level completion was low; they reached level 4 (out of 10) by the 1-month time point. At the 1-month assessment, participant A indicated that they enjoyed the weekly mood check-ins and that these were “eye opening” with regard to their symptoms.

#### Affective Engagement

At baseline, affective engagement measured by expectancy was high (80%). At the 1-month follow-up, affective engagement reflected by the exit questionnaire ratings (out of 7=“completely agree”) was excellent (satisfaction=6, helpfulness=7, and user-friendliness=7). At the 1-month assessment, participant A indicated that they liked the notifications and the ability to schedule and reschedule exercise sessions at their convenience.

#### Summary

Participant A was considered “Consistently low” as they did not reach an adherent level of use on a weekly basis, or cumulatively, throughout the month following discharge. Despite low use, participant A demonstrated moderate cognitive engagement and strong affective engagement. Therefore, we speculate that other factors may have affected their behavioral engagement. Notably, participant A’s month after discharge coincided with the onset of the COVID-19 pandemic. Participant A’s lack of activity in week 3 seemed to coincide with an increase in suicidality, for which they received a risk evaluation from the senior author. Their qualitative data revealed other life factors that increased their stress level during their transition out of acute care (ie, moving out of their parents’ home during the onset of the COVID-19 pandemic and conflict with family), which may have contributed to their low use.

### Participant B: “Adherent”

Participant B had a primary diagnosis of major depression, was living alone, and was preparing to apply to college. Participant B completed all follow-up assessments.

#### Behavioral Engagement

During the month after discharge, participant B completed 75% (3/4) of the weekly check-ins, as well as 3 self-initiated mood check-ins. Participant B was categorized as adherent as they completed 13 of the 12 suggested exercises.

#### Cognitive Engagement

At baseline, cognitive engagement, assessed by credibility ratings on a scale out of 9 (“completely”), was good (treatment logicality=6 and usefulness of treatment=7). During the 1-month postdischarge period, participant B completed 4 Habitdiary entries that covered several themes such as dating, current treatment, general mental health status, and awareness of improvement of symptoms. Level completion was good; they completed level 8 by the 1-month time point. At the 1-month assessment, participant B mentioned “[HabitWorks] allowed me to have more control over negative automatic thoughts.”

#### Affective Engagement

At baseline, affective engagement measured by expectancy was low, with the expected symptom improvement rated at 30%. In the daily sessions, participant B consistently reported finding the app easy to use. At the 1-month follow-up, affective engagement reflected by the exit questionnaire ratings (out of 7=“completely agree”) was excellent (satisfaction=6, helpfulness=6, and user-friendliness=7). In the qualitative interview, participant B said that they found the app easy to use and feasible to fit into the structure of the day.

#### Summary

Participant B was considered “Adherent” as they met the suggested exercise completion benchmarks. Despite their low expectancy early in treatment, they demonstrated strong behavioral, cognitive, and affective engagement throughout the month. They did not exhibit high initiation to use app features outside of the prompted occasions.

### Participant C: “Drop-off”

Participant C was a teacher and had a primary diagnosis of major depression. Participant C adhered to the study protocol through week 3 of the postdischarge month. Drop-off during week 4 coincided with the transition from remote to in-person learning at their school, and participant C subsequently did not complete the 1-month follow-up assessment.

#### Behavioral Engagement

During the month after discharge, participant C completed 50% (2/4) of the weekly check-ins, as well as 7 self-initiated mood check-ins, all before a drop-off in week 4. Exercise completion during the month after discharge (ie, 13) was adherent but reflected a drop-off in use; participant C completed 5 exercises in weeks 1 and 2, a total of 3 exercises in week 3, and no exercises in week 4.

#### Cognitive Engagement

At baseline, participant C’s cognitive engagement, assessed by credibility ratings (out of 9=“completely”), was low to moderate (treatment logicality=5 and usefulness of treatment=3). Participant C commented on having trouble with the WSAP and ambiguous situations related to work. Participant C completed 6 Habitdiary entries that covered several themes such as symptom improvement and current treatment, social functioning, work, and COVID-19–related worries (ie, getting COVID-19 at work and wearing a mask). Level completion was excellent; they reached level 10 by the end of week 3.

#### Affective Engagement

At baseline, affective engagement measured by expectancy was low, with expected symptom improvement rated at 10%. As participant C did not complete the 1-month follow-up, the exit questionnaire ratings and qualitative interviews could not be used to indicate the level of affective engagement.

#### Summary

Participant C was considered “Drop-off” as they initially exceeded the suggested number of exercises and then suddenly dropped off in use and did not complete the follow-up assessment. Although participant C was active, they used all app features (ie, diary, mood surveys, and exercises) and showed good cognitive engagement. Participant C’s drop-off coincided with the transition from remote to in-person school during the COVID-19 pandemic, and they had previously voiced concerns about this transition because of the fear of contracting COVID-19.

### Participant D: “High Diary”

#### Overview

Participant D had a primary diagnosis of panic disorder. Participant D was excited to participate and “contribute to science” and was attuned to the app, frequently reporting perceived glitches or malfunctions to study staff. Participant D stated that they wanted to be completely adherent and completed all study assessments.

#### Behavioral Engagement

During the month after discharge, participant D completed 100% (4/4) of the weekly check-ins, as well as 1 self-initiated mood check-in. Exercise completion during the postdischarge month was generally adherent, although slightly less than suggested (ie, 10): a total of 5 exercises in week 1, a total of 2 exercises in weeks 2 and 3, and 1 exercise in week 4.

#### Cognitive Engagement

At baseline, cognitive engagement, assessed by credibility ratings (out of 9=“completely”), was very good (treatment logicality=9 and usefulness of treatment=5). Participant D completed 11 Habitdiary entries and seemed to primarily use this feature as a tool for monitoring sleep, food, symptoms, and medication changes. Level completion was very low, remaining at level 1 by the end of the month after discharge. Despite not improving in exercise accuracy, participant D reported that it was “cool that [the app made me] notice my negative automatic thoughts” and that it was “eye-opening” in that it created greater awareness of interpretive style in daily life.

#### Affective Engagement

At baseline, affective engagement measured by expectancy was good, with expected symptom improvement rated at 70%. At the 1-month follow-up, affective engagement reflected by the exit questionnaire ratings (out of 7=“completely agree”) was good (satisfaction=6, helpfulness=5, and user-friendliness=6). In the qualitative interview, participant D reported that they liked the checklists to personalize stimuli and subsequently found all presented stimuli relatable.

#### Summary

Participant D was considered “High diary” as they clearly developed a preference for the Habitdiary feature. Indeed, although participant D completed 10 exercises during the postdischarge month, they seemed to use *HabitWorks* primarily for its diary function rather than connecting the WSAP exercises to their daily life. Similarly, they did not seem to benefit from the interpretation bias intervention exercises, as indicated by them never progressing beyond level 1 (indicating low interpretation accuracy).

### Participant E: “Super User”

#### Overview

Participant E had a primary diagnosis of major depression. Participant E was extremely interested in participating mentioning past positive experiences with mental health apps and an interest in continuing to use apps to address mental health concerns. Participant E was active throughout the study and completed all the study assessments.

#### Behavioral Engagement

During the month after discharge, participant E completed 100% (4/4) of the weekly check-ins, as well as 22 self-initiated mood check-ins. Exercise completion during the postdischarge month was extremely high (ie, 60 total, 15 exercises per week).

#### Cognitive Engagement

At baseline, cognitive engagement, assessed by credibility ratings (out of 9=“completely”), was moderate to good (treatment logicality=7 and usefulness of treatment=5). Participant E completed 17 Habitdiary entries, using this feature as intended to track negative automatic thoughts, as well as negative interpretations of events occurring in daily life. Themes present in the diary entries included worries about the future, romantic relationships, family, and health. Level completion was high, reaching level 10 by the end of the month after discharge. During the follow-up assessment, participant E reported that they found the situations personally relevant and noticed that handling some real-life situations was more challenging after they stopped using the app.

#### Affective Engagement

At baseline, affective engagement measured by expectancy was low, with the expected symptom improvement rated at 30%. At the 1-month follow-up, affective engagement reflected by the exit questionnaire ratings (out of 7=“completely agree”) was excellent (satisfaction=7, helpfulness=6, and user-friendliness=7). Throughout the study, participant E reported that the exercises were fun and enjoyable. In the 1-month qualitative interview, participant E reported that they enjoyed both the routineness (ie, consistent daily and weekly elements) and the “game component” of the app. They also mentioned sometimes struggling to quantify symptoms over the past 24 hours during weekly check-ins and sometimes found the app stimuli redundant.

#### Summary

Participant E was considered a “Super user” as they far exceeded benchmarks for exercise completion during the month after discharge. They also completed an extremely high number of Habitdiaries and user-initiated mood surveys during this period.

## Discussion

### Principal Findings

We examined patterns of behavioral engagement with a new mental health app designed to facilitate a healthier interpretive style as well as cognitive therapy skills practice following discharge from short-term psychiatric care. First, we operationalized engagement using a model that captures its multifaceted and dynamic nature and presented 5 cases reflecting the engagement patterns present in the sample. The data revealed heterogeneity across participants in behavioral use patterns, as well as variability within participants in their behavioral, cognitive, and affective engagement.

### Behavioral Engagement

We identified 5 patterns of engagement in our sample: consistently low, adherent, drop-off, high diary, and superuser. Most of the participants (22/31, 71%) were categorized as adherent or superuser. This finding differs from the typical pattern of quick disengagement with mental health apps. Indeed, only 16% (5/31) participants were categorized as consistently low in use. This may be because of the framing of the app as an augmentation and extension of care, motivation and excitement to use the app in our sample, and the engagement enhancement strategies used in *HabitWorks*.

High behavioral engagement may have been because of the use of bachelor’s degree–level staff for human support throughout the protocol [50,46]. *HabitWorks* is unique in that it shifts from a guided intervention (during acute care) to a fully automated or user-automated intervention (postdischarge period) [72]. However, even as a user-automated intervention, research staff played an important role, checking in with progress via weekly email, answering any technical or content-related questions regarding the app, and scheduling follow-up assessments. Notably, participant D mentioned the usefulness of staff in handling technical issues that arose, an issue area that often otherwise results in dropout [17].

The evidence supporting the usefulness of human support brings to the forefront the therapeutic alliance within app research, a well-documented, robust predictor of treatment outcome in traditional mental health care [73]. Human support may promote an alliance by creating step-by-step “process accountability” and enhancing agency and investment in treatment [16]. In *HabitWorks,* human support was delivered by research staff who checked in with the participants and monitored their app data (both exercise and mood scores) throughout the study. This type of support in *HabitWorks* cultivated a sense of “teamwork” among the app, staff, and participant, in essence, an alliance. As defined, the therapeutic alliance seems to subsume the aspects of affective (ie, expectancy and liking) and cognitive (ie, trust and credibility) engagement. Overall, our findings suggest that human support may have positively influenced behavioral engagement at various points throughout the study.

### Cognitive Engagement

Indicators of cognitive engagement varied across the 5 cases. Although cognitive engagement assessed by initial credibility ratings ranged from average to good, level completion varied dramatically across the cases. Level progression in *HabitWorks* required the achievement of 90% accuracy in the current level. We might expect practice, or exercise completion, to be associated with level achievement. However, participant D “High diary” completed 10 exercises after discharge but still did not progress past level 1. This is surprising, and one might conclude that participant D misunderstood the exercise, was inattentive during the exercise sessions, or was not engaged cognitively with the app.

However, in addition to level completion, cognitive engagement with *HabitWorks* was elicited by the Habitdiary function, which prompted participants to journal briefly about when they noticed themselves jumping to negative conclusions in their daily lives. Participant D (“High diary”) used the feature somewhat differently than the other participants (ie, as a free-response diary and self-monitoring record) and completed a high number of diary entries. Their qualitative data indicated that they were aptly applying the principles of the app to their life. Taken together, we may conclude that this participant showed a preference toward the diary feature and was in fact cognitively engaged, despite their lack of level progression. This apparent discrepancy may highlight the importance of measuring each facet of engagement with >1 indicator.

Qualitative data from all 5 cases added further nuance to our understanding of cognitive engagement, indicating that these participants found that the app helped them become aware of and assert control over their negative automatic thoughts, notice their interpretive style in their daily life, and better handle daily life situations. Participants’ use of CBT language (ie, negative automatic thoughts) in their feedback may illustrate a useful integration between the app and their CBT-based partial hospital treatment.

### Affective Engagement

Affective engagement, measured by expectancy for treatment to improve symptoms, was quite low for participants B, C, and E. However, at the 1-month assessment, all participants rated *HabitWorks* highly across acceptability indicators (ie, user-friendliness, satisfaction, and helpfulness). Qualitative feedback highlighted how participants easily integrated the app into their lives; how the app was relevant to their experiences; and that the app was fun, enjoyable, and game-like. Although it may be intuitive that a focus on subjective user experience is important to successful implementation [72], this focus may also be central to securing clinically meaningful benefits for users [21]. It is also notable that despite the initial low expectancy for some, all users ultimately reported enjoying the app. These findings suggest that *HabitWorks* has room for improvement in generating early “buy-in” in this population and support the conceptualization of affective engagement as a state that fluctuates over time.

### Relationships Between the Facets of Engagement

Although early affective engagement (ie, expectancy of app benefits) was low for some participants and high for others, these early ratings did not correspond in the expected direction with behavioral engagement throughout the 1 month. The typical relationship between expectancy and treatment engagement is such that lower expectancy is associated with lower engagement in treatment [74]. However, participant A had the highest expectancy and exhibited the lowest behavioral engagement, and participant E had low expectancy and exhibited the highest behavioral engagement. Moreover, all cases reported excellent affective engagement on the exit questionnaire. Although we cannot draw any conclusions from a case series, this observation underscores 2 aspects of the model of engagement by Nahum-Shani et al [27]: (1) engagement is dynamic and should be assessed in a corresponding manner and (2) the facets of engagement are related but distinct.

Participant C (“Drop-off”) illustrates the connection between cognitive engagement and behavioral engagement and the difficulty of relying on just one or the other to determine meaningful use. Although participant C’s use of the app suddenly dropped off after week 3 (ie, behavioral: shorter duration of use), they had already completed the prescribed number of exercises (ie, behavioral: adherent number of exercises) and had achieved the highest level possible in the app (ie, cognitive: interpretation bias accuracy). Their level completion indicates that they reached a “healthy” interpretation level (ie, 90% accuracy) at each level. Considering their behavioral and cognitive engagement together, we can surmise that they effectively used *HabitWorks,* suggesting that a drop-off in use is not necessarily problematic in all instances.

This discussion aligns with previous research suggesting that behavioral engagement alone does not necessitate better outcomes [22]. Indeed, some minimum amount of use may be necessary [75]; however, further use alone may not necessitate larger improvements. Similarly, participant A’s use pattern illustrates the proposition that sustained use may not be synonymous with meaningful use, and some participants may benefit from a period of inactivity. Specifically, participant A was inactive during week 3 but became active again later in the treatment month and went on to complete the 1-month assessment. Their period of disengagement may constitute a “recovery period,” a period of psychophysiological unwinding thought to be important to meaningful engagement [27], which allowed them to re-engage with the app subsequently. It is possible that this type of sporadic engagement may be a generally healthy or adaptive use style.

### Limitations and Future Directions

Our study had some limitations. First, the current case series included some indicators of engagement that were chosen post hoc and were specific to the *HabitWorks* app. Thus, it is difficult to compare engagement patterns across studies. Second, although in the RCT, *HabitWorks* was compared with an active control condition, differences in both features and recommendations for use between conditions prevented comparison of engagement patterns across conditions. Third, in our focus on incorporating strategies to maximize app use, it may be important to consider the potential for app overuse. We did not examine the length of the interaction time, which may be critical to further understanding effective use [76]. Problematic smartphone use that exceeds the necessary clinically meaningful use can become disruptive in the user’s life and lead to maintenance or furthering of psychosocial functioning impairment [77]. Fourth, the free-response diaries could be completed as desired by participants, and thus, the total amount of diary use varied across participants, with greater content available for those presumably more engaged in the app. Fifth, the categorization of participants was based solely on behavioral engagement. Future research with larger samples may apply quantitative analyses to identify more nuanced patterns of engagement that comprise all 3 facets, including cognitive and affective. Finally, it is possible that some engagement strategies were more helpful during early app interactions (ie, privacy and security), whereas others encouraged engagement during later interactions with the app (ie, novelty), and some others elicited engagement throughout (ie, human support). Given the conceptualization of engagement as state-like, it is likely that the helpfulness of these strategies was not linear. An important extension of this study will be to understand why users engaged with various app features [78] and which engagement strategies were the most helpful and at which time points.

### Conclusions

This case series of *HabitWorks* participants illustrated 5 patterns of engagement seen in our psychiatric sample transitioning out of CBT skills–based care. In the context of an RCT with specific recommendations for use and standardized delivery, 5 distinct patterns of engagement emerged. The study of engagement may be best approached from an individual difference’s perspective rather than with aggregated data. To better understand and promote “effective use” or “the extent, frequency, and duration of investment of physical, cognitive, and affective energies...to bring about a prespecified outcome” [27], a focus on multiple facets of engagement and their interactions may be important. This focus may ultimately allow for a better prediction of clinical outcomes.

## Appendix

Multimedia Appendix 1 Supplemental measures.

## Abbreviations

CBT: cognitive behavioral therapy
CEQ: Credibility and Expectancy Questionnaire
RCT: randomized controlled trial
REDCap: Research Electronic Data Capture
WSAP: Word Sentence Association Paradigm
