# Supplementary material for: Operationalizing Engagement With an Interpretation Bias Smartphone App Intervention: Case Series
Source: JMIR Ment Health. 2022 Aug 17;9(8):e33545. doi: 10.2196/33545 (PMC9434389; doi:10.2196/33545)
Supplement: Multimedia Appendix 1 [file mental_v9i8e33545_app1.docx]

**Multimedia Appendix 1**

1. **Exit Questionnaire**

1, Completely Disagree | 2, Moderately Disagree | 3, Slightly Disagree | 4, Neutral | 5, Slightly Agree | 6, Moderately Agree | 7, Completely Agree

1. I felt the HabitWorks smartphone app was helpful.

2. The sentences described situations that were relevant to me.

3. The HabitWorks smartphone app was easy to use (user-friendly).

4. I was satisfied with the HabitWorks smartphone app.

5. I would recommend the HabitWorks smartphone app to other people.

6. What do you think has changed for you because of the HabitWorks app?

7. What did you find most helpful about HabitWorks?

8. What did you find least helpful about HabitWorks?

9. Suggestions for improvement?

2. **HabitWorks Qualitative Exit Interview: Interview guide/question prompts**

1. General Impressions of HabitWorks app
2. What did you think about the HabitWorks app?
3. What did you find beneficial?
4. What was not helpful?
5. How could we improve the linkage of HabitWorks to your treatment?
6. What do you think about linking HabitWorks to your outpatient treatment?
7. Did you have any negative experiences due to the app?

B. Effect of Program

1. Do you feel like anything’s changed with you since you started the HabitWorks app?
2. Are you doing anything now or stopped doing anything as a result of the HabitWorks app?
3. Are you thinking about yourself or other people differently?
4. How can we improve the HabitWorks app?
